# The metabolic syndrome: a definition dilemma

**Published:** 2011-11

**Authors:** Arnab Ghosh

**Affiliations:** Department of Anthropology, Biomedical Research Laboratory, Visva Bharati University, Sriniketan, West Bengal, India

The metabolic syndrome (MS) is a common phenotype associated with an increased risk for type 2 diabetes mellitus (T2DM) and cardiovascular disease (CVD). Although there is no universally accepted definition for the MS, affected individuals commonly have a cluster of features, including abdominal obesity, hypertension, dyslipidaemia and dysglycaemia.1-4

The first formal definition of the MS was proposed by the World Health Organisation (WHO) in 1999. In the same year, the European Group for the Study of Insulin Resistance (EGIR) suggested a similar definition to that of the WHO, but excluded the microalbuminuria and diabetes components. In 2001, the United States National Cholesterol Education Program Adult Treatment Panel (NCEP ATP III) published a more practical definition for the MS, which eliminated insulin resistance as a criterion.1

A few years later, in 2005, the cut-off point for fasting plasma glucose was lowered, resulting in the modified NCEP ATP III (modified ATP III) definition.2 In 2005, the International Diabetes Federation (IDF) proposed a new definition for the MS, which made abdominal obesity, classified by ethnic-specific cut-off points, a necessary condition for the MS. In 2007, the IDF presented a definition of the MS for use in children and adolescents, thus becoming the first major organisation to do so.

Throughout the Asia-Pacific region, there are differences in the prevalence of obesity and metabolic disturbances. People of Indian origin (PIO) are ethnically a particularly vulnerable group from the standpoint of metabolic abnormalities.3 Keeping this in mind, researchers in the Indian diaspora are now using South Asian Specific (SAS) cut offs to define the MS in people of Indian origin.

The SAS definition of the MS is otherwise similar to the modified ATP III with the exception of cut offs for waist circumference (WC) (lower vs modified ATP III) and triglycerides (higher vs modified ATP III). However, it is noteworthy to mention that whether using the modified ATP III or SAS definition of the MS, a large number of individuals may be misclassified due to lack of a common minimum platform required to better comprehend the problem in people of Indian origin.3-5

Although widely used in epidemiological research, there has been ongoing concern that the WC cut-off points in the modified ATP III definition, which were predominantly intended for Americans, might not be appropriate for other ethnic groups, such as Asian Indians. South Asians (e.g. Asian Indians) have a more centralised distribution of body fat and a markedly higher mean waist–hip ratio (WHR) for a given level of body mass index (BMI) compared to Europeans.5 In Asian populations, morbidity and mortality is occurring in people with lower BMI and smaller WC. Therefore they tend to accumulate intra-abdominal fat without developing generalised obesity.4,5

Although the IDF definition of the MS in most instances failed to identify a subgroup of subjects who had the highest risk for CVD, before the IDF’s definition of the MS, the effect of ethnicity on the individual criteria for diagnosing the MS was not considered. Recently, comparisons of the prevalence of the MS between different ethnic groups have raised concerns about the validity of the WHO, NCEP ATP III, modified ATP III and IDF definitions when applied across different ethnic groups. The originally accepted criteria for the MS were based on risk prediction in non-Asian populations.6 However, recent data from the Asian population, including Asian Indians, indicate that these definitions may not be satisfactory for risk prediction.3

Although genetics most likely plays a crucial role in development of the MS, elucidating the exact genes involved has been hindered by the lack of a consistent definition of the MS,7 the varying combination of phenotypes even within a single definition, ethnic disparities, and gender influences. Recently, a large body of literature has emerged on early-life origins of the risk for the MS and associated diseases, such as coronary heart disease (CHD). Findings that experiences during the individual’s whole lifetime affect risk for disease highlight the need for an approach to understanding ethnic differences considering these early childhood conditions.

Moreover, research on the MS among children and adolescents and the implications of having the MS is limited. Because the roots of many adult chronic diseases originate in childhood, establishing a universally agreed definition of the MS in children and adolescents may help to identify children and adolescents who are at high risk for developing the MS, and allow for early prevention of possible adverse health events in early adulthood.

Differences in the prevalence of the MS and its components using the various definitions, both within and between populations, indicate that caution is required when comparing studies from different countries.7 Determining the clinical significance of these differences will require prospective outcome studies. Furthermore, to make the definition of the MS more sensitive, factors such as family history, habitual physical activity and smoking, along with region-specific cut offs for individual MS components are required to better comprehend the MS.4-7
